# Evaluation of the Applicability of Waste Rubber in Insulation Panels with Regard to Its Grain Size and Panel Thickness

**DOI:** 10.3390/ma17215251

**Published:** 2024-10-28

**Authors:** Zdravko Cimbola, Anđelko Crnoja, Ivana Barišić, Ivanka Netinger Grubeša

**Affiliations:** 1 Colas Hrvatska, Međimurska Street 26, 42000 Varaždin, Croatia; 2 Department of Construction, University North, 104 Brigade 3, 42000 Varaždin, Croatia; inetinger@unin.hr; 3 Faculty of Civil Engineering and Architecture Osijek, Josip Juraj Strossmayer University of Osijek, V. Preloga 3, 31000 Osijek, Croatia; ivana@gfos.hr

**Keywords:** waste rubber, porosity, modulus of elasticity, thermal properties, sound insulation, insulation panel

## Abstract

This paper explores the effect of waste rubber grain size on the porosity, modulus of elasticity, thermal properties, and soundproofing performance of polymer composites with different thicknesses (10, 15, and 20 mm). All properties were tested in accordance with European standards, with the exception of porosity, which was measured using Archimedes’ principle. The findings indicate that with a consistent amount of polyurethane glue, finer rubber grains result in composites with higher porosity, leading to a lower modulus of elasticity but enhanced thermal and sound insulation. In contrast, coarser rubber grains produced composites with lower porosity and a higher modulus of elasticity, though with slightly reduced thermal insulation and significantly worse soundproofing. A combination of fine and coarse rubber grains provided a balanced performance, offering both good thermal and sound insulation while maintaining a high modulus of elasticity. Among the thicknesses tested, 15 mm was identified as optimal, combining a relatively high modulus of elasticity, low thermal conductivity, and better airborne sound insulation index. Future research will focus on applying this composite in concrete building products that meet noise protection and energy efficiency standards.

## 1. Introduction

A healthy environment is fundamental to a healthy life. Science plays a key role in generating ideas and developing new principles that enable us to live more healthily and comfortably. To achieve this, it is essential to reduce the amount of waste and garbage disposed of in local landfills, which, despite protective measures, continue to pollute the environment to a degree that makes life unsustainable. One significant category of hazardous waste includes tires from cars, machinery, airplanes, and various other devices. According to ETRA [1,2], over 4700 tons of tires are produced annually worldwide, amounting to more than a billion units, with production and demand expected to rise even further. Beyond the recyclability of tires, an ongoing environmental threat comes from the wear and tear of these tires, which generates microplastic particles that infiltrate the environment due to their small size [3]. Promoting a circular economy is crucial, as it encourages innovative supply chains, resource conservation through recycling and re-manufacturing, and reduced pollution, which in turn helps lower carbon emissions [4]. Currently, efforts are focused on improving products derived from all components of waste tires.

Rubber granulate is a versatile raw material that can be combined with various materials for a wide range of applications. The authors highlight several possibilities for producing composite materials using rubber granulate in combination with wood and geopolymer binders [5]. Waste rubber is also widely explored in road construction sector, where its application in asphalt sector is well known [6,7,8], as well as in other bearing layers of pavement [9,10,11]. As the most used construction material, concrete containing waste rubber has been broadly investigated for different applications and purposes. Generally, adding rubber to concrete results in lower compressive strength, elastic modulus, and splitting tensile and flexural strengths due to a lack of proper bonding with the cement matrix and weak rubber strength, making it unsuitable for structural purposes [12,13]. Nevertheless, increased crumb rubber content results in concrete of lower unit weight, decreased water absorption, and increased total porosity, air voids, and ductility [14]. This creates potential for its application in non-structural properties such as thermal or acoustical panels since adding scrap rubber from waste tires results in an excellent soundproofing and thermal insulation properties [14,15,16,17,18]. As presented in [16], sound insulation properties can be improved by nearly 70% for concrete with 100% fine aggregate replacement by rubber particles.

The drawbacks of rubberized concrete create potential for research of waste rubber as a material in polymer composites for sound and thermal insulation panels. Rubber waste granulates combined with the matrix of elastomers or thermoplastics can be used to produce various kinds of new rubber–polymer with mechanical properties and performance depending on the type of used polymer matrix, the type and size of rubber grains, and their quantity in the composites [19]. Ground tire rubber may be incorporated into fresh polymer, but the result is typically lower mechanical properties due to insufficient bonding between rubber and the virgin matrix, but with improved thermal stability [20]. However, in [21], higher energy absorption and more deformation ability is observed for epoxy composites containing crumb rubber. In [22], polymer composites based on ground tire rubber and ethylene–vinyl acetate copolymer showed higher electrical conductivity attributed to the presence of carbon black in the rubber waste. For the same polymer composite, in [23], a long-standing flame retardancy is observed. As a limiting factor of wider application, in [24], emission of odorous volatile compounds and degradation products during thermomechanical processing of ground tire rubber or polymer/ground tire rubber systems was also observed. On the other hand, utilization of waste tire rubber as a filler for polymer composites noticeably reduces material costs [25].

This research aims to define the influence of waste rubber particle size on the physical (porosity), mechanical (modulus of elasticity), acoustical, and thermal properties of waste rubber polymer in order to produce sound and thermal insulation panels for non-structural purposes. Scope of this paper is to analyze rubber particle size’s influence on different properties in order to produce an environmentally and economically acceptable construction product.

## 2. Materials and Methods

### 2.1. Materials

**Figure 1:**
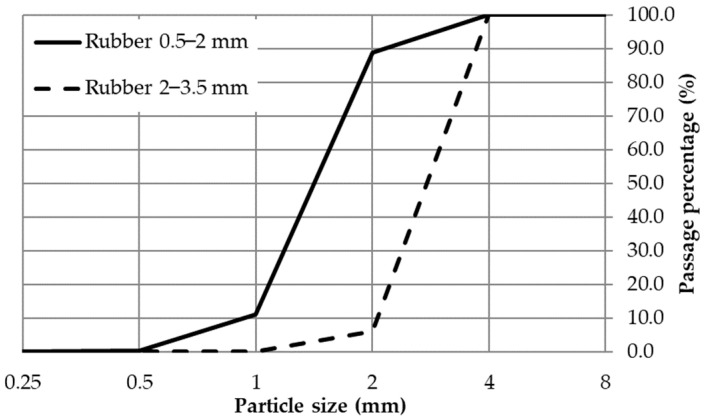
Grain size distribution of waste tire rubber.

**Figure 2:**
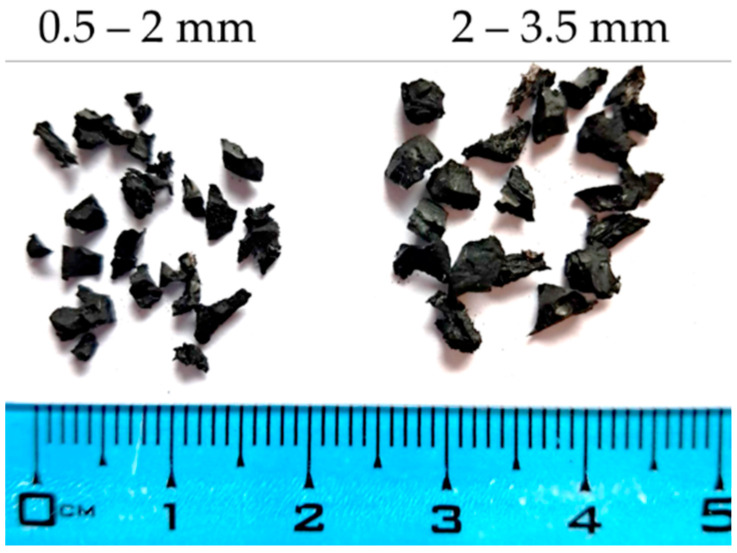
Image of granulated waste rubber.

Within this research, waste tire rubber and polyurethane glue densities of 1.2 g/cm^3^ were used to produce polymer composite samples to be tested as sound and thermal insulation panels. For this purpose, recycled car tires produced by mechanical recycling-cold method were used. This method included removing the tires’ edge reinforcements, followed by the tearing the tires into smaller pieces, suitable for a grinding machine. By grinding, the rubber is crushed into granules, which are separated according to different grain sizes. Within this research, rubber of two grain sizes was used: 0.5–2 mm and 2–3.5 mm. Also, a mixture of two fractions was used, consisting of 35% small fraction (0.5–2 mm) and 65% of coarse fraction (2.5–3 mm). Figure 1 and Figure 2 present the grain size distribution of the rubber used and its appearance, respectively.

Polyurethane-based glue with a density of 1.2 g/cm^3^ was used. The amount of binder was determined by the technological production process depending on aggregate granulometry and ranged from 465 g/m^2^ in mixes 1 to 6 to 858 g/m^2^ in mixes 7 to 9. Detailed compositions of the tested polymer composites are presented in Table 1.

Samples were produced by mixing polyurethane glue and waste rubber at 80 °C to 130 °C and pouring the mixture into the molds. Pressure was applied until a certain sample thickness was achieved. Samples of different sizes were produced depending on the tested property, as presented in Section 2.2.

### 2.2. Methods

#### 2.2.1. Porosity

The determination of porosity of polymer materials or materials made of recycled rubber is not standardized, and for this reason, standard “EN 993-1:2018: Methods of test for dense shaped refractory products—Part 1: Determination of bulk density, apparent porosity and true porosity” [26] was used. This standard defines sample dimensions to be in a range from 50 cm^3^ to 200 cm^3^. So, within this research, samples were made as 1 m^2^ panels, different in thickness, as presented in Table 1, from which test samples were cut out in dimensions of 5 × 10 × d mm, where “d” represents thickness, as defined in Table 1.

**Figure 3:**
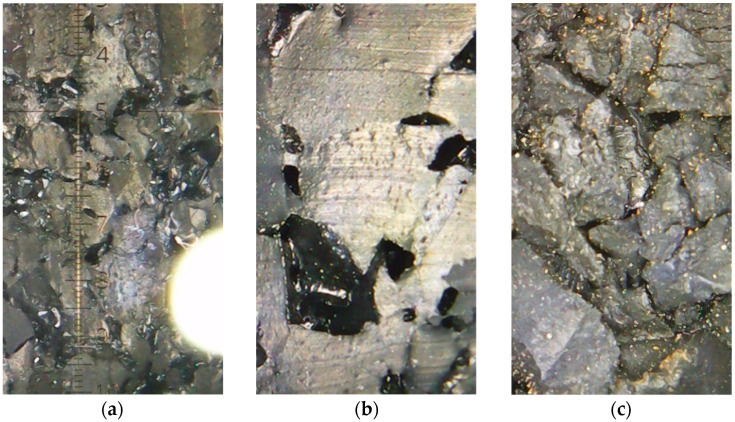
Cross-sections of 10 mm thick panels made from various rubber grain sizes: (**a**) 0.5–2 mm; (**b**) 2–3.5 mm; (**c**) 35% 0.5–2 mm + 65% 2–3.5 mm.

**Figure 4:**
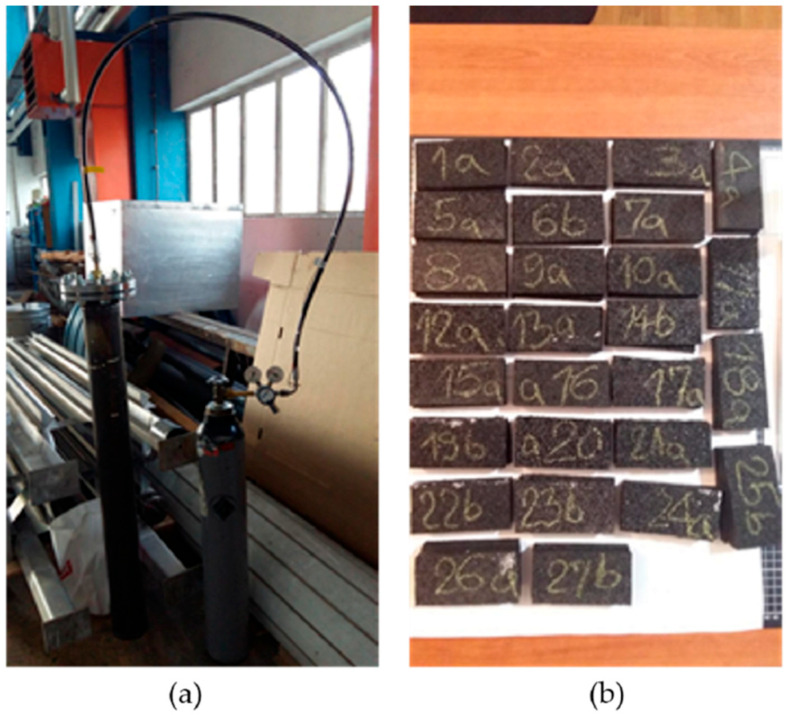
Porosity test setup (**a**) and used samples (**b**).

The test method for porosity determination used within this research was based on the connection between Archimedes’ law and the relationship between volume and mass of water. The basic goal is to determine the difference between a dry sample and a saturated sample that can no longer receive any water. Samples were exposed to high pressure in order to fill all pores with water, recording its mass change until constant mass was reached. The pressure used ranged from 16 to 20 bar. Mass recording was carried out every two days, and constant mass was reached after up to 8 days. In Figure 3, a sample cross-section taken by optical microscope is presented, where pores are seen as dark spots and the lighter parts are solid particles, while Figure 4 presents the test setup designed for the purposes of this research. The bright spot in Figure 3a is caused by the reflection from the microscope lens.

#### 2.2.2. Modulus of Elasticity

The panels’ mechanical properties were assessed regarding possible connections with other formwork, self-supporting of the panels, and resistance to exploitation. It is also important that such panels are resistant to various invasive effects, such as water, sound, light radiation, etc., which can affect the structure of the material and its decay. The binder inside the panel must be resistant to various actions, because its adhesive properties make the panel a stable structure.

**Figure 5:**
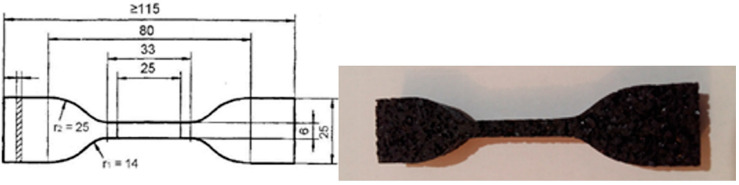
Modulus of elasticity test sample.

Modulus of elasticity was determined by subjecting samples to uniaxial tensile stress according to standard “EN ISO 527-4: 2022 Plastics—Determination of tensile properties—Part 4: Test conditions for isotropic and orthotopic fibre-reinforced plastic composites” [27]. The test samples were cut by thin stream of water under high pressure from the produced panels in the dimensions presented in Figure 5. Modulus of elasticity was calculated for stress and deformation values of min 20% lower than the yield point (the border between the elastic and plastic areas).

#### 2.2.3. Thermal Conductivity Measurement

The main methods by which thermal conductivity is determined are the classical methods of stationary one-dimensional heat flow through a plate or cylinder with guard rings. The idea of these methods follows from the basic Fourier conduction law, which establishes a direct proportional dependence of the heat flux from the temperature gradient. One of the simplest methods for determining thermal conductivity is the method of M. Povalyaev [28], which is theoretically based on the laws of the non-stationary temperature field, which were developed in the papers of Lykov and Mihajlov [29] and Chudnovsky [30]. A feature of the method is to take into account the heat content of the sensor using an empirical coefficient. The absence of accounting for lateral heat losses is eliminated by introducing a correction factor ψλ = f(ψ0). The advantage of the method is its short experiment duration of 2–4 min and a small temperature difference of 1–2 °C. The gain limit was selected at 0.05 mV. The minimum division value during measurement is 0.002 mV, which allows for taking responses with an accuracy of 0.001 mV. To ensure tight contact of thermocouple junctions with the sample surface, the sensors are clamped in the measuring cassette using a special device. The time between measurements for each sample was 30, during which the sample was thermally stabilized so that the previous experiment would not affect the following one.

**Figure 6:**
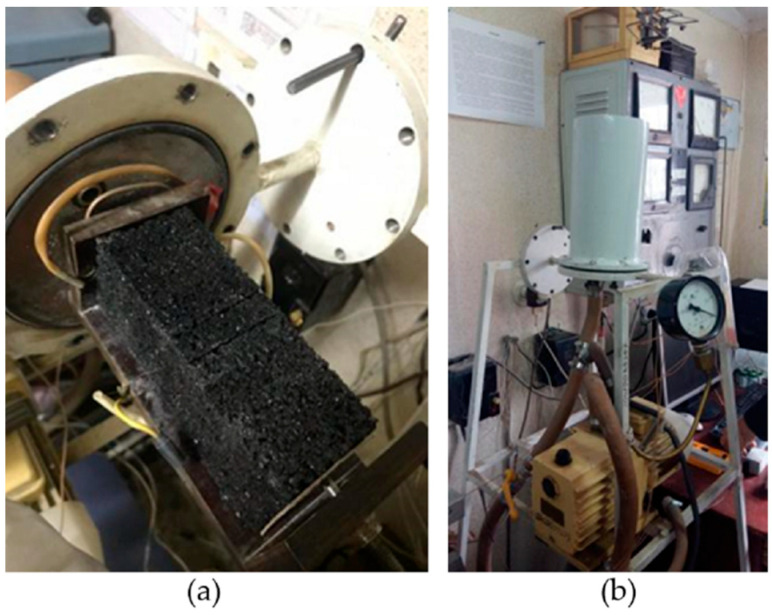
Thermal conductivity test setup: (**a**) measuring cassette with a sample; (**b**) general view of test setup.

Samples were cut from molded beams (Figure 6) in dimensions of 50 × 50 × d (mm), where “d” is the thickness of the sample (panel), either 10, 15, or 20 mm, as presented in Table 1. The main requirement for samples is the maximum possible parallelism of large faces, as well as compliance with the flatness of the faces.

#### 2.2.4. Airborne Sound Insulation Index

**Figure 7:**
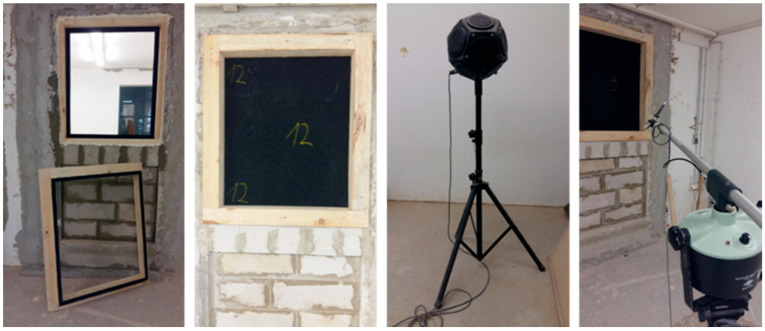
Airborne sound insulation index test setup.

The sample was tested as window glass in a window element in a frame specially designed for this research in dimensions 1 × 0.765 × d (m) where “d” is a thickness of the sample (panel), 10, 15, or 20 mm, as presented in Table 1. The conditions for the frame are airtightness and good sealing of all working holes. Figure 7 shows the airborne sound insulation index test setup. The implementation procedure is according to the standard “EN ISO 717-1:2020: Acoustics—Rating of sound insulation in buildings and of building elements—Part 1: Airborne sound insulation” [31]. The test sample is located in a double partition wall (the walls are dilated—the test room is designed as a room in a room) between the sound-receiving area and the sound source area. Inside the sound source area, there is a non-directional sound source, while in the receiving room, there is a very sensitive Class I microphone on a rotating stand. The rotation takes place in an inclined plane in such a way as to register different pressure levels in the reception area and any other influences that may occur during the test. The emitted sound of the sound source is approximately 105 dB for a duration of 60 s.

## 3. Results and Discussion

### 3.1. Porosity

**Figure 8:**
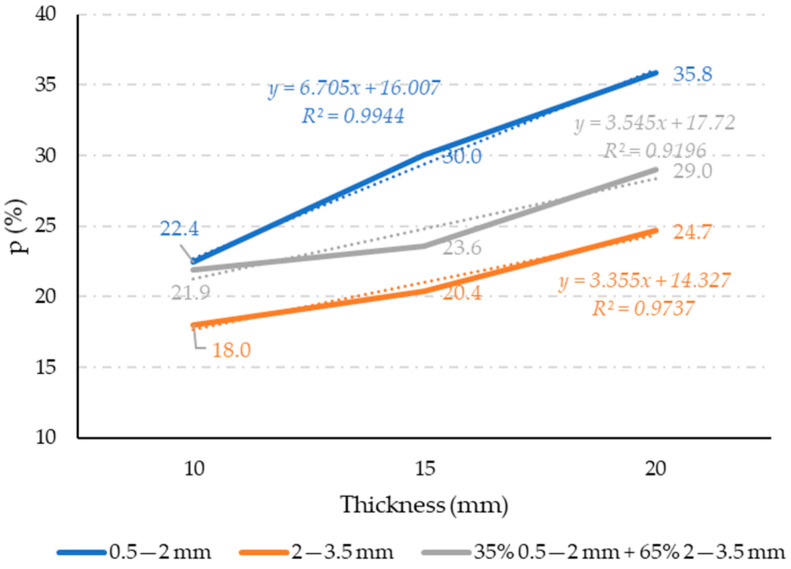
Results of porosity measurement.

The measured porosity results are shown in Figure 8, with each value representing the mean of three measurements for each property. The standard deviations are up to 20%.

As visible out of Figure 8, with the increase in rubber grain size, porosity decreases in a nearly ideal linear relation. The decrease in porosity with the increase in rubber grain size may be attributed to higher specific surface area value of fine grain size (0.5–2 mm) compared to coarse grain size (2–3.5 mm) [32]. The smaller rubber grains have a larger specific surface area, and when the same amount of glue is used across all rubber fractions for panels of equal thickness, the coverage of the smaller grains with glue is less effective, leading to greater porosity. The fine rubber fraction produces the highest porosity, while the coarse fraction results in the lowest porosity. The porosity of mixtures containing both fine and coarse rubber falls between these extremes. These findings align with Figure 3, which illustrates a high proportion of small pores in panels made from fine rubber (Figure 3a), a lower proportion of larger pores in panels made from coarse rubber (Figure 3b), and a combination of both small and large pores in panels composed of mixed rubber fractions (Figure 3c). In a way, the fine rubber particles acted as an air bubbles in the polymer matrix. This phenomenon is evidenced by the more sensitive change in porosity with panel thickness increase for finer-grain-size rubber particles. Namely, the regression line for fine-grain-size rubber particles has a higher slope with the increase in panel thickness, which indicates a greater influence of panel thickness on such composites compared to a coarse rubber grain size.

Panel thickness also affects porosity. Although mixtures 1 to 6 maintain a consistent glue-to-rubber ratio (Table 1) and would be expected to exhibit similar porosity across panels of different thicknesses, Figure 8 shows that porosity increases with panel thickness. However, this increase can be considered a statistical error, as the results shown have an accuracy of up to 20%. In mixtures 7 to 9, where the glue-to-rubber ratio is significantly higher than in mixtures 1 to 6 (Table 1), a decrease in porosity with increasing thickness was expected, yet the opposite occurred. This suggests that the process for pressing panels is more efficient for thinner panels than for thicker ones.

### 3.2. Modulus of Elasticity

**Figure 9:**
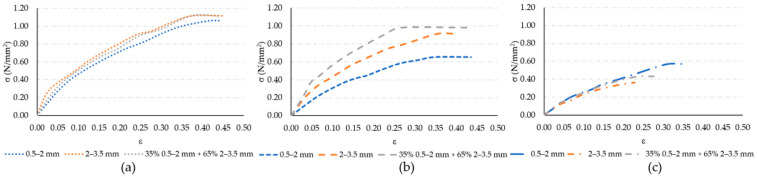
σ–ε diagrams of polymer composite samples: (**a**) 10 mm thick; (**b**) 15 mm thick; (**c**) 20 mm thick.

**Figure 10:**
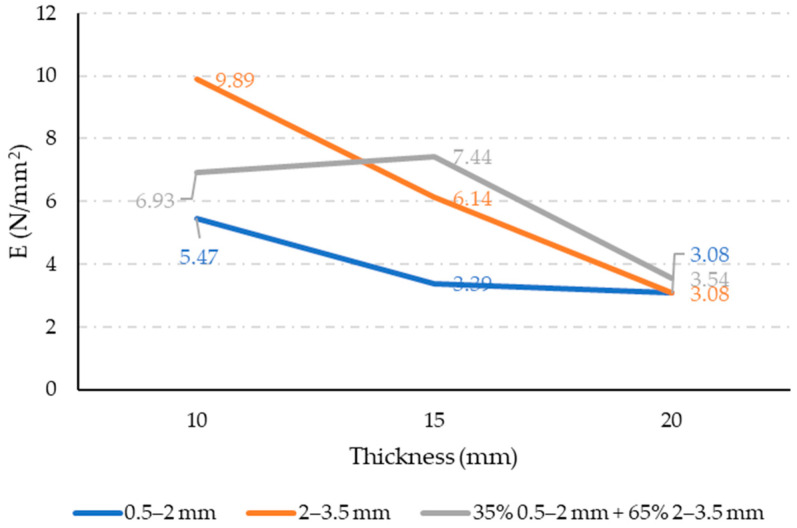
Results of modulus of elasticity measurement.

The σ–ε diagrams for the test specimens sawn out of each panel are shown in Figure 9, with the curve representing the average of three recorded measurements. The standard deviation was up to 20%. Based on the diagrams in Figure 9, the modulus of elasticity under tension was calculated within the deformation range of 0.05–0.25%, as specified by the standard “ISO 527-1:2019 Plastics—Determination of tensile properties—Part 1: General principles” [33]. The calculated modulus of elasticity results are shown in Figure 10. Presented results indicate high influence of both observed parameters: rubber grain size and panel thickness. The fine rubber particles resulted in high decrease in modulus of elasticity, indicating the higher deformability of such mixtures. This is in line with the result of increased porosity for such mixtures. On the other hand, for coarser grains, a significant influence of the panel thickness on the modulus of elasticity is observed. It indicates that for such a production method, the optimal panel thickness may be 15 mm. The combination of finer and coarser waste rubber grains exhibited the highest modulus of elasticity for a panel thickness of 15 mm, which is expected due to the effective interweaving of small and large granules, as seen in Figure 3c. Considering that the porosity results shown in Figure 8 do not suggest any reason for a reduced modulus of elasticity in the sample sawn out of a 10 mm thick panel made from a combination of fine and coarse rubber granules, the slightly lower obtained modulus of elasticity (6.93 N/mm^2^) in this sample can be attributed to a statistical error.

### 3.3. Thermal Conductivity

**Figure 11:**
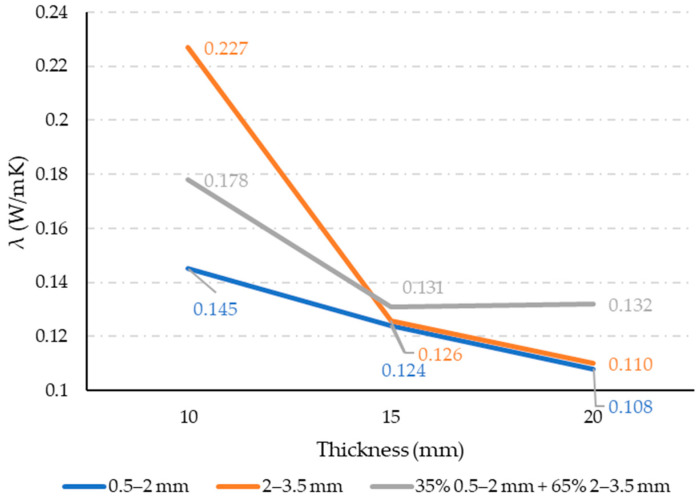
Thermal conductivity results.

The measured thermal conductivity results are shown in Figure 11, with each value representing the mean of three measurements for each property. The standard deviations are up to 20%. Measured thermal conductivity ranges from 0.1 W/mK to 0.2 W/mK, which is in line with data presented in [16], where thermal conductivity of rubber–epoxy composites is 0.25 W/mK, which is comparable to other non-structural materials such as foamed concrete (0.7 W/mK) or gypsum boards (0.17–0.23 W/mK), the most common material used as false ceilings and partition walls. For insulating materials, low conductivity is preferable. So, according to the presented results, fine rubber particles resulted in the lowest thermal conductivity. This will produce the most effective thermal insulation panel. This result lines in a line with porosity measurement. Materials with high porosity make for good insulation due to the presence of air in their pores acting as an additional insulator.

Generally, increasing panel thickness results in lower thermal conductivity, which presents a more effective insulation panel. The influence of panel thickness increase on the drop in thermal conductivity is less sensitive for fine-grain-size rubber particles compared to coarse grain mixtures.

N panels containing coarse grains, stable thermal flux is harder to be achieve due to a non-homogeneous material nature. In fine-grain-size mixtures, waste rubber particles are embedded in glue, creating a more homogenous structure, which enables stable thermal flux to be reached more easily.

### 3.4. Airborne Sound Insulation Index

**Figure 12:**
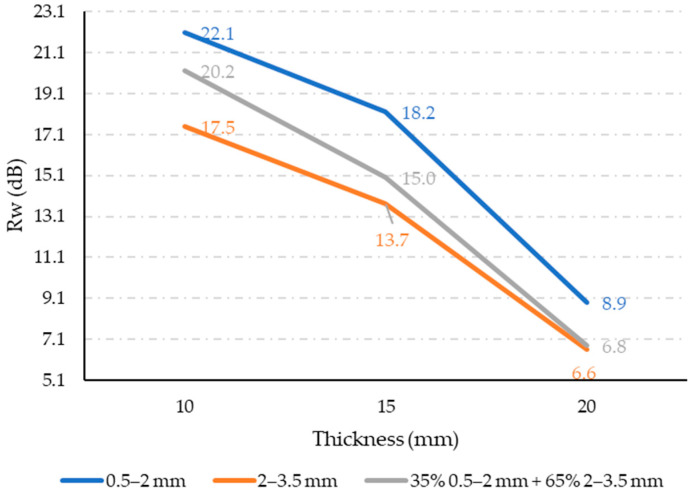
Sound insulation index measurement results.

The measured airborne sound insulation index results are shown in Figure 12, with each value representing the mean of three measurements for each property. The standard deviations are up to 20%. Our measurements show that samples with a fine structure exhibit the best sound properties, while those with a coarse structure, characterized by larger particles, perform the worst. These findings align with theoretical assumptions about airborne sound transmission. For sound insulation coverings, it is crucial to prevent the formation of air cavities in the materials, as these cavities can facilitate sound propagation. Fine-structured materials have smaller pores and a more uniform distribution of granulate throughout the cross-section, unlike coarse-structured materials (Figure 3a,b). In coarse structures, the larger particles create shorter pathways for airborne sound to travel through, allowing for sound to pass through the material more easily. Also, the trends here are very similar to those of the porosities presented in Figure 8.

### 3.5. Possible Application of Waste Rubber Panels in Civil Engineering

**Figure 13:**
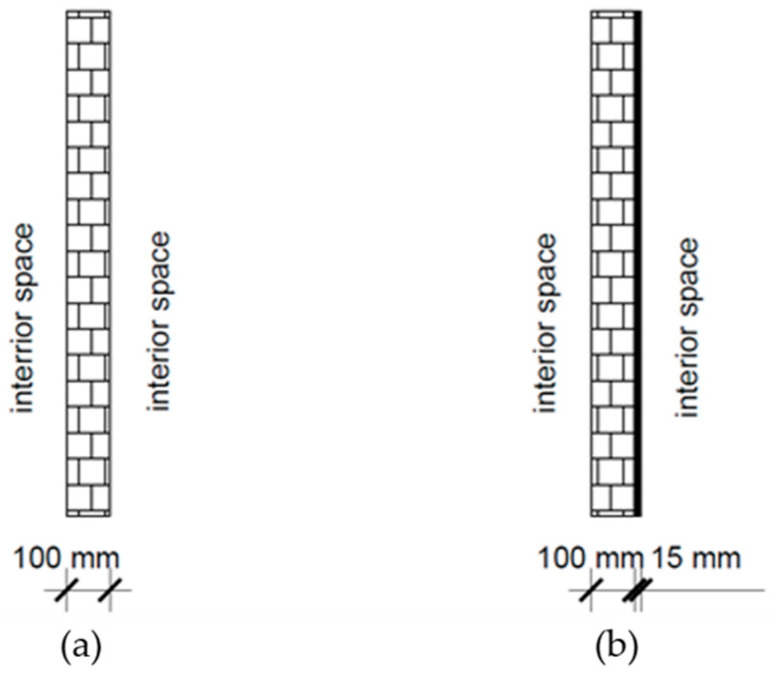
Application of waste rubber panels as sound and thermal insulation: (**a**) brick wall 100 mm; (**b**) brick wall 100 mm + recycled rubber 15 mm.

This section discusses the potential use of recycled rubber panels in civil engineering for the purpose of noise protection and enhancing a building’s energy efficiency. The effectiveness of these panels was analyzed by comparing an internal masonry wall (made of Porotherm units) without a waste rubber panel (Figure 13a) to one with a waste rubber panel (Figure 13b). The ability of the waste rubber panel to reduce noise and improve energy efficiency was evaluated. Given the relatively high modulus of elasticity, good sound insulation properties, and low thermal conductivity of the 15 mm thick panel made from a combination of fine and coarse rubber grains, this specific panel was selected for further calculations.

The effect of waste rubber panels on sound insulation was evaluated using a simplified calculation. For an internal masonry wall without a waste rubber panel (Figure 13a), Equation (1) was used [34]:

R_w_ = 20 × log_10_ × (m × f) − C (1)

For an internal masonry wall with a waste rubber panel (Figure 13b), Equation (2) was used [34]:

R_w_ = 20 × log_10_ × ((m_1_ + m_2_) × f) − C (2)

In both equations, the parameters are defined as follows: “R” is a weighted sound reduction index in dB; “m” is a mass of the masonry wall per unit area in kg/m^2^ (a value of 70 kg/m^2^ is adopted in this calculation); “f” is a sound wave frequency in Hz (a value of 500 Hz is adopted in this calculation); “m_1_” and “m_2_” are masses of the two wall layers in kg/m^2^ (in this research, m_1_ = 70 kg/m^2^ for the masonry wall and m_2_ = 11.25 kg/m^2^ for the waste rubber 15 mm thick panel); and “C” is a constant that depends on the characteristics of the wall and joints (a value of 47 is adopted in this research).

Using Equations (1) and (2), for an internal masonry wall without a waste rubber panel (Figure 13a), the weighted sound reduction index is calculated to be R_w_ = 43.88 dB, while for an internal masonry wall with a waste rubber panel (Figure 13b), weighted sound reduction index is calculated to be R_w_ = 45.18 dB. The calculation shows a 1.3 dB or 3% improvement in the sound insulation index when a 15 mm thick waste rubber panel is added to the masonry wall.

The effect of waste rubber paneling on the energy efficiency of an externally reinforced concrete wall was analyzed using EnCert-HR3 v.3.09 software. This is a free software program used for the energy certification of buildings and creation of projects for the rational use of energy and thermal protection of buildings that complies with all relevant European regulations. The following parameters were considered for masonry wall: specific heat capacity of 920 J/kgK, thermal conductivity coefficient of 0.33 W/mK, density of 700 kg/m^3^, and water vapor diffusion resistance factor of 1.0 for wall thickness of 100 mm. For the 15 mm thick waste rubber panel, the parameters were as follows: specific heat capacity of 1400 J/kgK (not measured, but based on the values available for rubber), thermal conductivity of 0.131 W/mK, density of 750 kg/m^3^, and water vapor diffusion resistance factor of 150. The result of the heat transfer coefficient through the building element for an internal masonry wall without a waste rubber panel (Figure 13a) is U_w_ = 2.11 W/m^2^K, while for an internal masonry wall with a waste rubber panel (Figure 13b), U_w_ = 1.7 W/m^2^K. The calculation of energy efficiency implies a significant reduction in thermal energy loss, approximately 20% when using recycled rubber paneling as thermal insulation.

## 4. Conclusions

This paper investigated the impact of waste rubber grain size on the porosity, modulus of elasticity, thermal properties, and soundproofing performance of polymer composites of varying thicknesses (10, 15, and 20 mm). The results achieved suggest that with a consistent amount of polyurethane glue, a finer rubber grain size produces polymer composites with higher porosity (and consequently a lower modulus of elasticity) but offers better heat and sound insulation. Conversely, using a coarse rubber grain size resulted in lower porosity (and thus a higher modulus of elasticity), but slightly poorer thermal insulation and significantly worse soundproofing. A mixture of both fine and coarse rubber grain size provided the composite with balanced thermal and sound insulation properties while maintaining a high modulus of elasticity. Regarding composite thickness, 15 mm was found to be optimal, offering a relatively high modulus of elasticity, low thermal conductivity, and improved airborne sound insulation. The researchers plan to continue the research in the form of the application of this composite in concrete building products that meet the requirements of noise protection and energy efficiency.

## Figures and Tables

**Table 1:** Polymer composite composition.

Mix	Sample Thickness (mm)	Rubber Grain Size (mm)	Glue Content (kg per 1 m^3^)	Rubber Content (kg per 1 m^3^)	Glue-to-Rubber Ratio (%)	Density (kg/m^3^)	Panel Mass (kg/m^2^)
1	10	0.5–2	46.5	1053.50	4.41	1100	11.0
2	10	2–3.5	46.5	1053.50	4.41	1100	11.0
3	10	0.5–2 (35%) + 2–3.5 (65%)	46.5	1053.50	4.41	1100	11.0
4	15	0.5–2	31.67	718.33	4.41	750	11.25
5	15	2–3.5	31.67	718.33	4.41	750	11.0
6	15	0.5–2 (35%) + 2–3.5 (65%)	31.67	718.33	4.41	750	11.0
7	20	0.5–2	42.90	542.10	7.91	585	11.7
8	20	2–3.5	42.90	542.10	7.91	585	11.0
9	20	0.5–2 (35%) + 2–3.5 (65%)	42.90	542.10	7.91	585	11.0

## Data Availability

Dataset available on request from the authors.
